# Supplementary figures and images for: Neofunctionalization of a second insulin receptor gene in the wing-dimorphic planthopper, Nilaparvata lugens
Source: PLoS Genet. 2021 Jun 28;17(6):e1009653. doi: 10.1371/journal.pgen.1009653 (PMC8270448; doi:10.1371/journal.pgen.1009653)

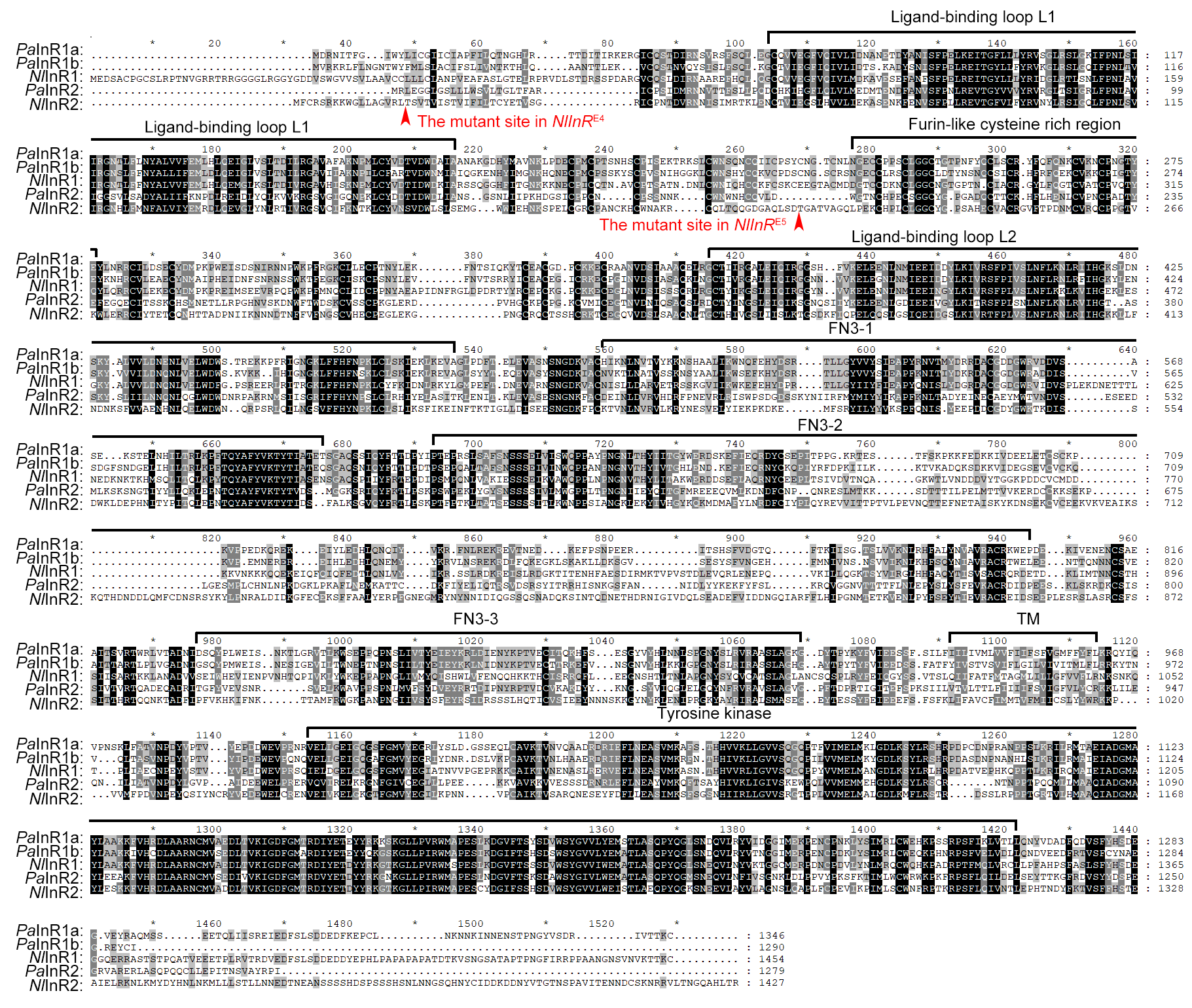

Supplement: S1 Fig — Conserved domains of two ligand-binding loops (L1 and L2), a furin-like cysteine-rich region, three fibronectin type 3 region, a single transmembrane (TM), and a tyrosine kinase region were indicated. The Cas9 cutting sites for NlInRE4 and NlInR2E5 mutants were indicated by arrowheads. PaInR1a and PaInR1b, the linden bug Pyrrhocoris apterus InR1 homologue (GenBank: KX087103.1 and KX087104.1). PaInR2, P. apterus InR2 homologue (GenBank: KX087105.1). NlInR1 and NlInR2, the brown planthopper Nilaparvata lugens InR1 (GenBank: KF974333.1) and InR2 (GenBank: KF974334.1) homologue, respectively. (TIF) [file pgen.1009653.s001.tif]

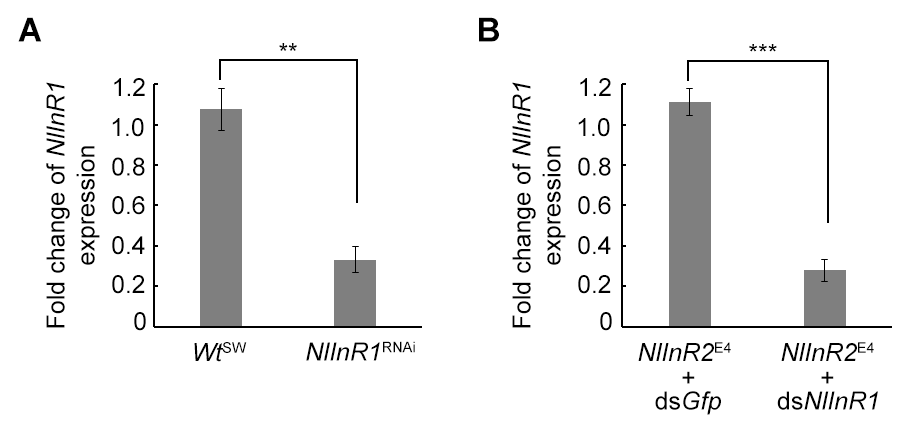

Supplement: S2 Fig — (TIF) [file pgen.1009653.s002.tif]

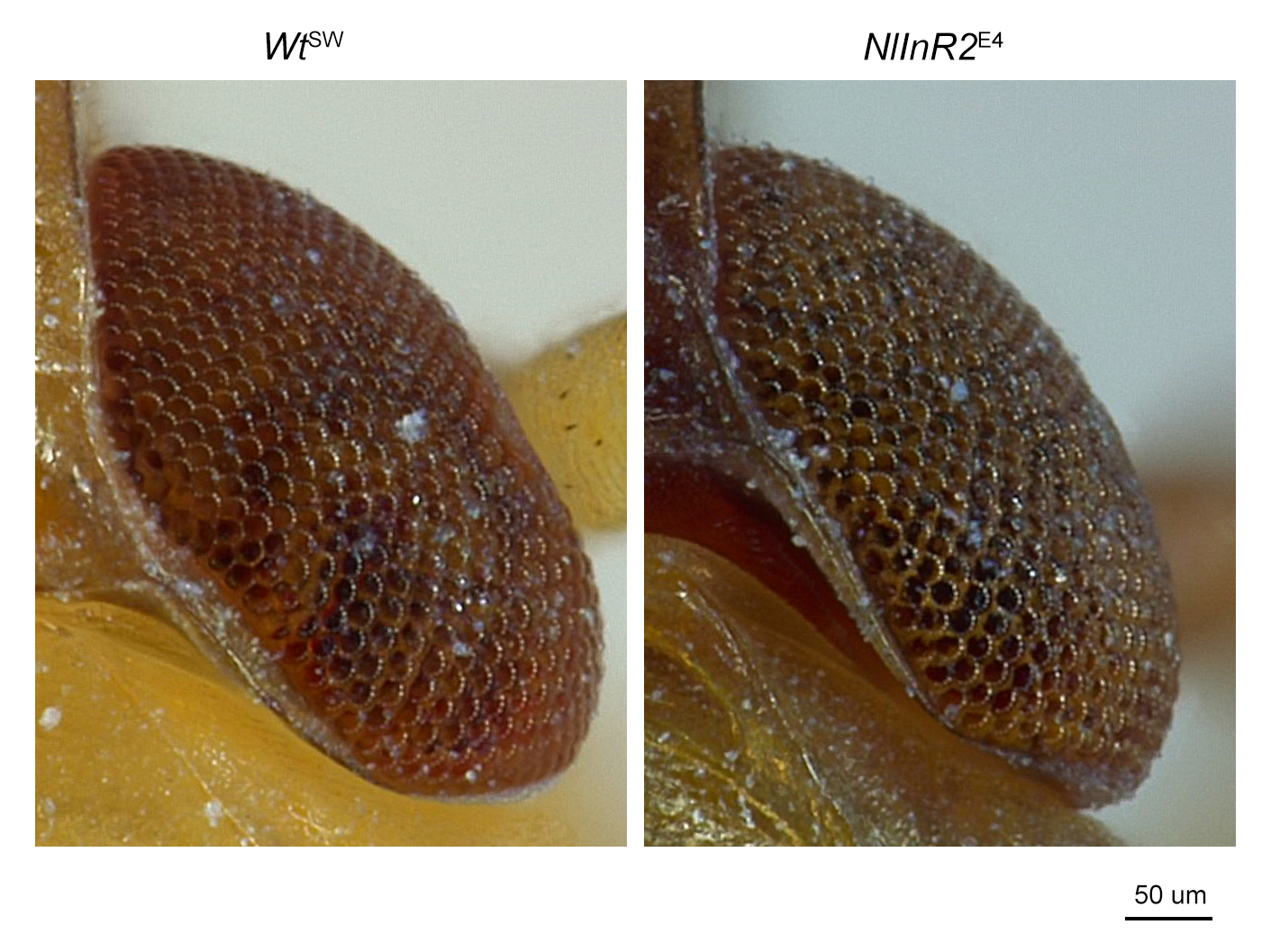

Supplement: S3 Fig — 48h-4th-instar WtSW or 12h-5th-NlInR2E4 nymphs were microinjected with dsNlInR1 or dsGfp. BPHs (n = 5 for each of three replicates) at 24 h after adult eclosion were collected, and the NlInR1 expression was examined in the context of for WtSW (A) and NlInR2E4 (B) by qRT-PCR. The relative expression of NlInR1 was normalized to the expression level of rps15. Statistical comparisons were performed using a two-tailed Student’s t-test (**, P < 0.01 and ***, P < 0.001), and bars represent mean ± s.e.m. (TIF) [file pgen.1009653.s003.tif]

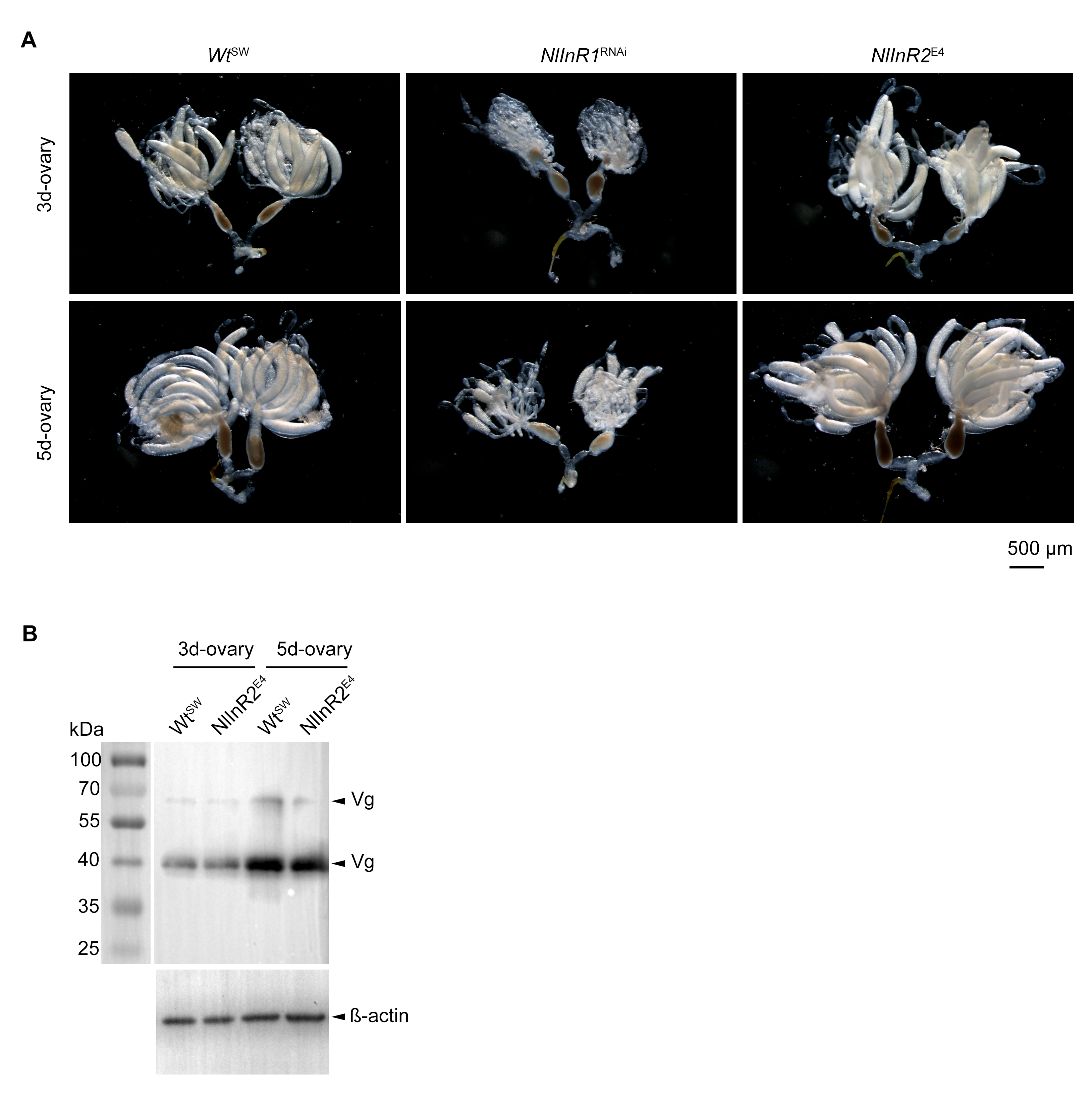

Supplement: S4 Fig — (A) Ovaries were dissected from WtSW, NlInR1RNAi, and NlInRE4 at 3 and 5 days after adult eclosion. NlInR1RNAi was derived from 5th-instar nymphs microinjected with dsNlInR1. (B) Western blotting assay of Vg in ovaries. Ovaries were immunoblotted with anti-Vg polyclonal antibody. The antibody against ß-actin was used as a loading control. (TIF) [file pgen.1009653.s004.tif]

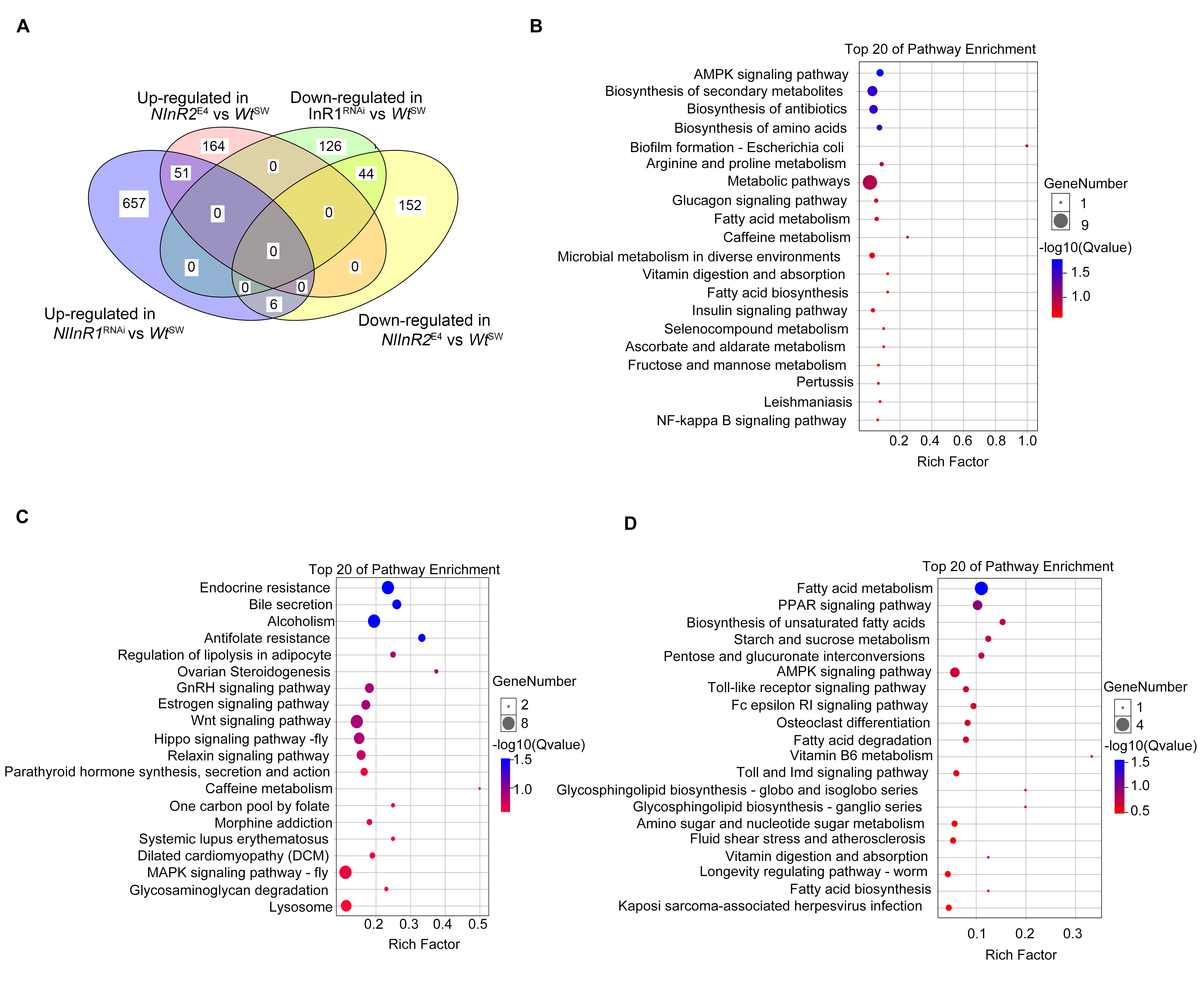

Supplement: S5 Fig — (A) The number of differentially expressed genes (DEGs) in NlInR1RNAi and NlInR2E4 females compared to WtSW. (B) Top 20 enriched Kyoto Encyclopedia of Genes and Genomes (KEGG) pathways of common DEGs regulated by NlInR1RNAi and NlInR2E4. (C) Top 20 enriched KEGG pathways of DEGs specifically regulated by NlInR1RNAi. (D) Top 20 enriched KEGG pathways of DEGs specifically regulated by NlInR2E4. (TIF) [file pgen.1009653.s005.tif]
