# Supplementary material for: Neofunctionalization of a second insulin receptor gene in the wing-dimorphic planthopper, Nilaparvata lugens
Source: PLoS Genet. 2021 Jun 28;17(6):e1009653. doi: 10.1371/journal.pgen.1009653 (PMC8270448; doi:10.1371/journal.pgen.1009653)
Supplement: S1 Data — (DOCX) [file pgen.1009653.s026.docx]

**S1 Data**

**Transcriptomic analysis of wing buds in *NlInR2*^E4^ and *Wt*^SW^ BPHs**

**Overview of the RNA-seq data.** Fifth-instar *NlInR2*^E4^ and *Wt*^SW^ nymphs at 24- and 48-hAE were collected, and then T2W and T3W were dissected for RNA sequencing (RNA-seq). We constructed 24 cDNA libraries from 8 samples with three biological replicates each. For each cDNA library, at least 31 million raw reads were generated via Illumina Hiseq platform with 0.02% sequencing error rate (S1 Table). After removing low-quality reads, adaptor, and unknown base (N) reads, at least 31 million clean reads were produced from each cDNA libraries (S1 Table). The mapping rate of clean reads against *N. lugens* reference genome ranged from 65.58% to 70.97%. To assess the reproducibility among three biological replicates in each sample, the pairwise correlations between every two samples in the same group was calculated. A high Pearson correlation coefficients (R^2^ > 0.92) was detected among three biological replicates, indicating the RNA-seq data was reliable.

**Comparison of transcriptional profiles between** **InR2_24h_T2W and WT_24h_T2W.** Based on the criteria of fold-change ≥ 2 and adjusted *P* (padj) < 0.01, we identified 1,846 (9.96%, 1,846 out of 18,534 genes) DEGs in T2W of 24h-5^th^-instar *NlInR2*^E4^ (InR2_24h_T2W) compared to that of *Wt*^SW^ (WT_24h_T2W), including 706 up-regulated and 1140 down-regulated genes (S2 Table). GO enrichment analysis showed that the up-regulated 706 DEGs were significantly enriched into 682 GO terms with false discovery rate (FDR) < 0.05, including 529 terms in biological process category, 111 terms in cellular component category, and 42 terms in molecular function category. The top 20 significantly enriched GO terms of up-regulated DEGs were mainly involved in cell proliferation, such as cell cycle process (GO:0022402, 124 DEGs), cell cycle (GO:0007049, 135 DEGs), DNA metabolic process (GO:0006259, 89 DEGs), mitotic cell cycle (GO:0000278, 104 DEGs), and chromosome organization (GO:0051276, 111 DEGs) (S3 Table). The down-regulated 1140 DEGs were enriched into 61 GO terms, including 31 terms in biological process category, 12 terms in cellular component category, and 18 terms in molecular function category. Most of them were involved in extracellular matrix component and cuticle development, such as extracellular region (GO:0005576, 81 DEGs), cuticle development (GO:0042335, 41 DEGs), structural constituent of chitin-based cuticle (GO:0005214, 20 DEGs), intrinsic component of membrane (GO:0031224, 91 DEGs), and steroid metabolic process (GO:0008202, 14 DEGs) (S4 Table).

**Comparison of transcriptional profiles between InR2_24h_T3W and WT_24h_T3W.** Compared to T3W of 24h-5^th^-instar *Wt*^SW^ nymphs (WT_24h_T3W), T3W of 24h-5^th^-instar *NlInR2*^E4^ nymphs (InR2_24h_T3W) had 1542 DEGs (8.32%, 1,542 out of 18,534 genes), the number is comparable to DEGs in *NlInR2*^E4^ T2W versus *Wt*^SW^ T2W. InR2_24h_T3W up-regulated 638 genes and concomitantly down-regulated 904 genes with fold-change ≥ 2 and padj < 0.01 (S5 Table). The up-regulated DEGs were significantly enriched into 668 terms (FDR < 0.05), including 490 terms in biological process category, 130 terms in cellular component category, and 48 terms in molecular function category. Similar to T2W, the up-regulated 638 DEGs in InR2_24h_T3W versus WT_24h_T3W were mainly associated with cell cycle, such as cell cycle (GO:0007049, 128 DEGs), cell cycle process (GO:0022402, 114 DEGs), chromosome organization (GO:0051276, 111 DEGs), mitotic cell cycle (GO:0000278, 95 DEGs), and chromosome (GO:0005694, 96 DEGs) (S6 Table). The down-regulated 904 DEGs in InR2_24h_T3W vs WT_24h_T3W were significantly assigned to 90 GO terms, including 29 terms in biological process category, 16 terms in cellular component category, and 45 terms in molecular function category (S7 Table). Analogous to T2W, the most down-regulated DEGs were significantly enriched to GO terms related to extracellular matrix and cuticle development. Taken together, the RNA-seq data from both T2W and T3W of nymphs at 24 hAE supports the accelerated cell proliferation in *NlInR2*^E4^ wings.

**Comparison of transcriptional profiles between InR2_48h_T2W and WT_48h_T2W.** We identified 2,245 DEGs in T2W of 48h-5^th^-instar *NlInR2*^E4^ nymphs (InR2_48h_T2W) versus *Wt*^SW^ nymphs (WT_48h_T2W) with fold-change ≥ 2 and padj < 0.01, including 1,185 up-regulated and 1,060 down-regulated DEGs (S8 Table). Judged by FDR < 0.05, the up-regulated and down-regulated DEGs were assigned to 33 and 381 GO terms, respectively. The up-regulated DEGs were significantly enriched into GO terms associated tissue patterning and morphogenesis such as extracellular region (GO:0005576, 58 DEGs), membrane (GO:0016020, 160 DEGs), epidermis development (GO:0008544, 16 DEGs), hair cell differentiation (GO:0035315, 14 DEGs), and imaginal disc-derived wing hair organization (GO:0035317, 13 DEGs) (S9 Table). This phenomenon differs the up-regulated DEGs in T2W of *NlInR2*^E4^ at 24 hAE, where most of GO terms are associated with cell proliferation (S3 Table). Additionally, majority of down-regulated DEGs were associated with DNA replication and cell cycle, including cell cycle process (GO:0022402, 114 DEGs), DNA replication (GO:0006260, 49 DEGs), DNA metabolic process (GO:0006259, 82 DEGs), chromosome (GO:0005694, 98 DEGs), and DNA-dependent DNA replication (GO:0006261, 42 DEGs) (S10 Table). This evidence indicate that the expression level of cell proliferation-associated genes decreases as *NlInR2*^E4^ nymphs proceed from 24 hAE to 48 hAE, and *NlInR2*^E4^ may start to prepare for nymph-adult metamorphosis.

**Comparison of transcriptional profiles between InR2_48h_T3W and WT_48h_T3W.** A total of 1,055 up-regulated and 1,531 down-regulated DEGs were identified in T3W of 48h-5^th^-instar *NlInR2*^E4^ (InR2_48h_T3W) versus *Wt*^SW^ (WT_48h_T3W) nymphs with fold-change ≥ 2 and padj < 0.01 (S11 Table). The up-regulated DEGs were significantly enriched into 53 GO terms with FDR < 0.05, which included 31 terms in biological process, 16 terms in cellular component and 6 terms in molecular function. Similar to T2 wing buds at 48 hAE, the up-regulated DEGs of InR2_48h_T3W versus WT_48h_T3W were enriched to GO terms of tissue patterning and morphogenesis (S12 Table). The down-regulated DEGs of InR2_48h_T3W versus WT_48h_T3W were significantly enriched into 352 terms with FDR < 0.05, including 252 terms in biological process, 74 terms in cellular component and 26 terms in molecular function. We found most of down-regulated DEGs were significantly enriched to cell proliferation, such as cell cycle process (GO:0022402, 111 DEGs), DNA replication (GO:0006260, 41 DEGs), DNA-dependent DNA replication (GO:0006261, 34 DEGs), cell cycle (GO:0007049, 125 DEGs), and mitotic cell cycle (GO:0000278, 91 DEGs) (S13 Table). These data indicates that both *NlInR2*^E4^ T2W and T3W might stop cell proliferation, and commence wing morphogenesis at 48 hAE.
