# Supplementary material for: Neofunctionalization of a second insulin receptor gene in the wing-dimorphic planthopper, Nilaparvata lugens
Source: PLoS Genet. 2021 Jun 28;17(6):e1009653. doi: 10.1371/journal.pgen.1009653 (PMC8270448; doi:10.1371/journal.pgen.1009653)
Supplement: S2 Data — (DOCX) [file pgen.1009653.s027.docx]

**S2 Data**

**Transcriptomic analysis of *NlInR2*^E4^ and *NlInR1*^RNAi^ female adults**

**Overview of transcriptomes.** Because *Nl*InR2 resembled *Nl*InR1 on fecundity, but differed from *Nl*InR1 on lifespan and starvation tolerance at the adult stage, we used RNA-seq to examine genome-wide gene expression in *Wt*^SW^, *NlInR2*^E4^, and *NlInR1*^RNAi^ females. For this purpose, fourth-instar nymphs were microinjected with ds*NlInR1* to generate *NlInR1*^RNAi^ females. At 12h after adult eclosion, females (*n* = 8) were pooled for RNA extraction, and a total of nine cDNA libraries were constructed from *Wt*^SW^, *NlInR2*^E4^, and *NlInR1*^RNAi^ females with three biological replicates each. Then, cDNA libraries were further used for Illumina sequencing via Illumina Hiseq platform. More than 20.9 million raw reads were generated from each cDNA library with Q20 and Q30 values each exceeding 92.77% and 97.32% (S16 Table), respectively. Following the removal of adaptors, poly-N and low-quality reads, more than 20.1 million clean reads were retained for each sample. The mapping rate of clean reads against *N. lugens* reference genome ranged from 64.12% to 66.87%. The transcriptomic data was deposited into GenBank under the accession number PRJNA724037.

**Differentially expressed genes (DEGs) in *NlInR1*^RNAi^ and *NlInR2*^E4^ females.** Using the fold change ≥ 2 and FDR< 0.05 as criteria, we identified 884 DEGs in *NlInR1*^RNAi^ and 417 DEGs in *NlInR2*^E4^ compared to *Wt*^SW^ females (S5 Fig), which only accounted for 4.8% and 2.2% of BPH encoding genes (18,534 genes), respectively. Among all the DEGs identified, 101 genes (S17 Table) were commonly regulated by *NlInR1*^RNAi^ and *NlInR2*^E4^, of which six genes were up-regulated in *NlInR1*^RNAi^ but down-regulated in *NlInR2*^E4^, 51 genes were up-regulated in both *NlInR1*^RNAi^ and *NlInR2*^E4^, and 44 genes were down-regulated in both *NlInR1*^RNAi^ and *NlInR2*^E4^ (S5 Fig). Kyoto Encyclopedia of Genes and Genomes (KEGG) pathway analysis showed that the 101 commonly-regulated DEGs were significantly involved into metabolic pathways (S5 Fig). In addition, 783 (S5 Fig and S18 Table) and 316 (S5 Fig and S19 Table) genes were specifically regulated by *NlInR1*^RNAi^ and *NlInR2*^E4^, respectively. KEGG pathways enriched by genes of *NlInR1*^RNAi^-specific were visibly different from that enriched by genes of *NlInR2*^E4^-specific (S5 Fig). These observations indicate that *Nl*InR1 and *Nl*InR2 may have different sets of target genes.
