# Supplementary material for: Neofunctionalization of a second insulin receptor gene in the wing-dimorphic planthopper, Nilaparvata lugens
Source: PLoS Genet. 2021 Jun 28;17(6):e1009653. doi: 10.1371/journal.pgen.1009653 (PMC8270448; doi:10.1371/journal.pgen.1009653)
Supplement: Attachment — Submitted filename: Response Letter.docx [file pgen.1009653.s028.docx]

**Point-to-point respond to reviewers**

**Respond to reviewers #1:**

**Q1：** *Reviewer #1: Major opinion and suggestion.*

*The main argument of this manuscript is that in BPH, InR1 and InR2 are functionally different in largely two points; 1. Life history aspect, and 2. Wing polymorphism. InR2 knockout affected nymph development duration and fecundity. This manuscript used previous report [reference 28] where phenotype of InR1 knockdown by RNAi is demonstrated, to compare with InR2 knockout phenotype. I think for this manuscript to gain more originality and novelty, it should include more detailed mechanism of how each InR1 and InR2 work differently. This manuscript did a good job in finding distinct phenotypes of InR2 knockout, so there should be distinct mechanism of InR2 gene action compared to InR1 which leads to different functions. In terms of wing polymorphism, detailed mechanism of InR1 and InR2 is already reported [reference 28], and this manuscript reemphasized this previous argument by showing increased cell number and increased mRNA expression coding for cell cycle and DNA replication related pathway in InR2 knockout wing disc. Evolution of insect insulin receptor in different insect species is already investigated in reference 27. Therefore, I am suggesting that focusing more on comparison of life history aspect of each InR1 and InR2 knockout (or knockdown), and identifying molecular mechanisms to explain this consequence will improve novelty of this manuscript. Is InR2 able to transduce ILP signal? Does InR2 have different ILP ligand than InR1? What is the function of InR2 in ovary or fat body to regulate fecundity? I believe answering such questions will further support the title of this manuscript ‘Functionally independent evolution of a second insect insulin receptor gene’.*

**Respond to Q1：**Reviewer #1 raised an important question on the mechanism of how each InR1 and InR2 work differently. Although we don’t have direct evidences to resolve this question, the following three experiments we did might provide some clues:

**Experiment #1: *Nl*InR2 was co-precipitated with itself by immuneprecipitation assay.**

Because the insulin receptor is a dimeric glycosylated receptor tyrosine kinase, we asked whether *Nl*InR2 could form *Nl*InR2-*Nl*InR2 dimer in BPH. To this end, we constructed *Nl*InR2-V5 and *Nl*InR2-Flag recombinant plasmids, and then co-transfected into *Drosophila* S2 cells to express V5- or Flag-tagged *Nl*InR2 fusion proteins. Subsequently, we used anti-V5 or anti-Flag antibodies to do co-immunoprecipitation assay. Our results showed that *Nl*InR2-V5 was successfully co-precipitated with *Nl*InR2-Flag (**Rebuttal Letter Fig. 1**), indicating that *Nl*InR2 might form homo-dimers in BPH.

In addition, a noticeable difference between *Nl*InR1 and *Nl*InR2 sequences is that four cysteine (C) residues were present in the N terminus of the furin-like cysteine-rich region (Fu) of *Nl*InR1, but absent in *Nl*InR2 (**Rebuttal Letter Fig. 1B**). We asked whether these four cysteine residues contribute InR1-InR1 formation. For this purpose, we point mutated each cysteine to alanine (C-to-A) to construct recombinant plasmids *Nl*InR1^4A^-V5 and *Nl*InR1^4A^-Flag. Then, *Drosophila* S2 cells were transfected with *Nl*InR1^4A^-V5 and *Nl*InR1^4A^-Flag to express V5- or Flag-tagged *Nl*InR1^4A^ fusion proteins. Co-immunoprecipitation assay with anti-V5 or anti-Flag antibodies showed that *Nl*InR1^4A^-V5 was co-precipitated with *Nl*InR1^4A^-Flag (**Rebuttal Letter Fig. 1C**), indicating that these four cysteine residue might be not necessary for *Nl*InR1-*Nl*InR1 dimer formation.

**Rebuttal Letter Fig. 1. Immuneprecipitation assay of *Nl*InRs. (A)** *Drosophila* S2 cells were transfected with *Nl*InR2-V5 and *Nl*InR2-Flag recombinant plasmids to express V5- or Flag-tagged *Nl*InR2 fusion proteins. Co-immunoprecipitation assay was conducted using anti-V5 or anti-Flag antibodies. **(B)** Schematic diagram of domain structure of InRs. Four cysteine residues were present in *Nl*InR1, but not in *Nl*InR2. A signal peptide, two ligand-binding loops (L1 and L2), a furin-like cysteine-rich region, three fibronectin type 3 region, a single transmembrane (TM), and a tyrosine kinase region were shown. **(C)** V5- or Flag-tagged *Nl*InR1^4A^ fusion proteins were expressed in *Drosophila* S2 cells, and then used for co-immunoprecipitation assay with anti-V5 or anti-Flag antibodies. *Nl*InR1^4A^, *Nl*InR1 with cysteine to alanine mutations.

**Experiment #2: Comparative transcriptome analysis of *Wt*^SW^, *NlInR2*^E4^, and *NlInR1*^RNAi^ females at 12 h after adult eclosion.**

Because *Nl*InR2 resembles *Nl*InR1 on fecundity, but differs from *Nl*InR1 on lifespan and starvation tolerance at the adult stage, we used RNA-seq to examine genome-wide gene expression in *Wt*^SW^, *Nl*InR2^E4^, and *NlInR1*^RNAi^ females. The transcriptomic data was deposited into GenBank under the accession number PRJNA724037. Using the fold change ≥ 2 and FDR< 0.05 as criteria, we identified 884 differentially expressed genes (DEGs) in *NlInR1*^RNAi^ vs *Wt*^SW^ females and 417 DEGs in *NlInR2*^E4^ vs *Wt*^SW^ females (**Rebuttal Letter Fig. 2A**), which only account for 4.8% and 2.2% of BPH encoding genes (18,534 genes), respectively. Among all the DEGs identified, 101 genes were commonly regulated by *NlInR1*^RNAi^ and *NlInR2*^E4^, of which six genes were up-regulated in *NlInR1*^RNAi^ but down-regulated in *NlInR2*^E4^, 51 genes were up-regulated in both *NlInR1*^RNAi^ and *NlInR2*^E4^ , and 44 genes were down-regulated in both *NlInR1*^RNAi^ and *NlInR2*^E4^ (**Rebuttal Letter Fig. 2A**). Kyoto Encyclopedia of Genes and Genomes (KEGG) pathway analysis showed that the 101 commonly regulated DEGs were significantly involved into metabolic pathways (**Rebuttal Letter Fig. 2B**). In addition, 783 and 316 genes were specifically regulated by *NlInR1*^RNAi^ and *NlInR2*^E4^ (**Rebuttal Letter Fig. 2A**), respectively, and KEGG pathways enriched by genes of *NlInR1*^RNAi^-specific (**Rebuttal Letter Fig. 2C**) were different from that enriched by genes of *NlInR2*^E4^-specific (**Rebuttal Letter Fig. 2D**). These observations indicate that *Nl*InR2 and *Nl*InR2 may have different sets of target genes.

**Rebuttal Letter Fig. 2. Comparative transcriptome analysis of *Wt*^SW^, *NlInR2*^E4^, and *NlInR1*^RNAi^ females at 12 h after adult eclosion. (A)** The number of differentially expressed genes (DEGs) in *NlInR1*^RNAi^ and *NlInR2*^E4^ females compared to *Wt*^SW^. **(B)** Top 20 enriched Kyoto Encyclopedia of Genes and Genomes (KEGG) pathways of common DEGs regulated by *NlInR1*^RNAi^ and *NlInR2*^E4^. **(C)** Top 20 enriched KEGG pathways of DEGs specifically regulated by *NlInR1*^RNAi^. **(D)** Top 20 enriched KEGG pathways of DEGs specifically regulated by *NlInR2*^E4^.

**Experiment #3: Ovary morphology and vitellogenin (Vg) expression in ovaries of *NlInR2*^E4^.**

Given *NlInR2*^E4^ resembles *NlInR1*^RNAi^ on decreased fecundity, we examined the ovaries of *NlInR1*^RNAi^ and *NlInR2*^E4^ females at 3 and 5 days after adult eclosion. We noticed that *NlInR2*^E4^ had ovaries morphologically similar to Wt^SW^, while *NlInR1*^RNAi^ had immature ovaries (**S4 Fig**). In addition, western blot analysis showed that *NlInR2*^E4^ had a comparable level of vitellogenin (Vg) in ovaries relative to *Wt*^SW^ (**S4 Fig**). These observations indicate that *NlInR2* might regulate fecundity in a way different from *NlInR1*.

This new data have been added to the revised manuscript, S4 Fig and lines 202-207 in the combined manuscript_PG_clean copy.

**Experiment #4:** Our previous study (Xue et al., 2020, Insect Molecular Biology 29) showed that four ILPs had redundant and distinct roles in physiological processed in the BPH. In order to investigate whether *Nl*InR2 binds to specific ILPs, we aimed to do binding assay for InR and ILPs using purified *Nl*InR2 proteins and artificially synthesized ILPs. Unfortunately, we failed to purify *Nl*InR2 proteins when *Drosophila* S2 cells were used to express *Nl*InR2**.** On the other hand, we still question the activity of artificially synthesized ILPs even if we could get purified *Nl*InR2.

**Experiment #5:** We provided new data showing that (1) *NlInR2*^E4^ slightly and significantly reduced phallus length (Fig 1G), but had ovipositors with the size comparable to *Wt*^SW^ (Fig 1H), and (2) *NlInR2*^E4^significantly reduced starvation tolerance (Fig 2G) in contrary to an increased starvation tolerance in *NlInR1*^RNAi^.

This new data have been added to the revised manuscript, lines 176-179 and 211-213 in the combined manuscript_PG_clean copy.

*Minor questions.*

**Q2:***1. InR2 where is the functional domain? Since the sgRNA target exon 4 and 5, is there any possibility that truncated protein is still active?*

**Respond to Q2:** The domain construction of *Nl*InR1 and *Nl*InR2 was shown in S1 Fig. We designed a single guide RNA (sgRNA) for CRISPR/Cas9-mediated mutagenesis of *NlInR2*, which was located at 36 to 54 nucleotide (nt) downstream of the *NlInR2* start coden (ATG) in exon 4 (Fig 1A and S1 Fig, lines 159-162 in the combined manuscript_PG_clean copy). The sgRNA target exon 5 was located at the N terminus of furin-like cysteine rich region of InRs (S1 Fig). Both mutations should result in a complete dysfunction of *Nl*InR2 protein (S1 Fig).

**Q3:** *2. Fig 1. Scale bar missing in in D and E. Especially in Fig 1E, I assume the size of WT and NlInR2E4 is different.*

**Respond to Q3:** The Fig 1E in the original manuscript showed forewings of *Wt*^LW^ and *NlInR2*^E4^. Yes, forewings of *NlInR2*^E4^ were 17% smaller than that of *Wt*^LW^ (Fig 1B). The Fig 1E was replaced by Fig 1I in the revised manuscript, which showed both forewings and hindwings to answer reviewer #2’s questions.

**Q4:** *3. Fig 4. Fifth instar NlInR2E4 nymphs at 12 hAE were microinjected… But in the text in line 187, it is mentioned that 24h5th-instar was microinjected. Which one is true?*

**Respond to Q4:** We have corrected this mistake. 12h-5^th^-instar *NlInR2*^E4^ nymphs were microinjected with ds*Nl*InR1, lines 246-247 in the combined manuscript_PG_clean copy. It’s worth mentioning that the same phenotype was derived from either 12h or 24h 5^th^-instar *NlInR2*^E4^ nymphs was used for microinjection.

**Q4:** *4. The title is too ambitious. It would be more informative if title included the name of the species studied (BPH).*

**Respond to Q4:** The title has been changed to “Neofunctionalization of a second insulin receptor gene in the wing-dimorphic planthopper, *Nilaparvata lugens*”, as suggested by reviewer #2.

**Q5:** 5. InR2 mutant has increased cell cycle and number in wing disc. Is this effect restricted to wing disc? If this effect is wing disc specific, what is the explanation for this phenomenon?

**Respond to Q5:** Our previous study showed that *NlInR2* had a higher expression in wing buds by qRT-PCR analysis. In addition to wing buds, relative lower level of *NlInR2* was detected in other body parts, so *Nl*InR2 might have alternative function in addition to the role in wing development. However, available evidences we have now only indicate that knockdown of *NlInR1* and *NlInR2* led to SW and LW through common signaling elements of the insulin signaling cascade. We mentioned this in the revised manuscript, lines 135-143 in the combined manuscript_PG_clean copy.

**Respond to reviewers #2:**

*Reviewer #2: SUMMARY: Thank you for giving me the opportunity to review this manuscript by Xue et al. This manuscript examines the potential functional significance of a second insulin receptor in the brown plant hopper (BPH). Previous work using RNAi had established that InR1-RNAi caused the formation of miniature short-winged morphs (SW) with extended life span yet decreased fertility and compromised carb and lipid metabolism. In contrast InR2RNAi had no such consequences and instead shifted development to long winged morphs (LW). This was the first indication of functional divergence between InR paralogs in an insect. The existence of multiple paralogs in insects is generally common, though absent in Drosophila and C. elegans, and hence much understudied. In the present ms the authors expand on these earlier findings by employing CRISPR to create two different null mutants for InR2. They document that (a) InR2 null mutants mad minimal effect on fuel metabolism and life span (in contrast to what has been documented in flies and worms) (b) induce wing growth through (c) increased cell proliferation associated with (d) the upregulation of genes linked to cell growth and proliferation and (e) wing patterning genes. More generally the manuscript deepens understanding of the functional divergence between two InR paralogs in plant hopper development, and is likely to motivate many follow up studies in other non-model insect species.*

*SUMMARY EVALUATION: The ms is well written, thorough, and detailed. It addresses an important topic - because only a single InR exists in the by far most studied insect, our understanding of potential functional divergences of multiple InR paralogs in insect development and evolution is very modest, even though having more than one InR is typical of almost all insects. While the ms does not document evidence for functional divergence between paralogs in BPH for the first time, it significantly expands our understanding thereof, using technologies (CRISPR) that mark a major advancement in methodology for insect evo devo in hemimetabolous insects. All combined I view PLoS Genetics as an appropriate venue for this ms. Below I detail several major and minor comments that I hope the authors will find of use.*

*SIGNED: Armin Moczek*

**Q1：***MAJOR COMMENTS:*

*It was not clear to me (a) what the variation in body size was that existed among individuals within treatment groups, and whether efforts were made to correct for that variation in the statistical analyses. For example, larger female adults generally produce more eggs in most insects, so if WtSW animals are on average a bit bigger than NIInR2E4 animals (as seems to be the case from Fig. 2 B) then the difference in fecundity reported in figure 2 may just be a function of body size. Same goes for the metabolic data: glucose and triglyceride trend non-significantly to be less abundant in Wt animals, but if their relatively greater size is factored in this difference may become significant.*

**Respond to Q1:** We provided following new data to resolve this question:

(*1*) *NlInR2*^E4^ had hind tibia length (Fig 1D), head size (Fig 1E) and compound eyes (S2 Fig) comparable to *Wt*^SW^, indicating that *NlInR2*^E4^ had a similar body size to *Wt*^SW^ controls (**lines 170-172 in the combined manuscript_PG_clean copy**).

(*2*) Wild-type LW BPH (*Wt*^LW^) had a slighter heaver body mass than *Wt*^SW^ (Fig. 2B) because the former had larger wings. However, *Wt*^LW^ laid comparable numbers of eggs as *Wt*^SW^ (Fig 2E), which differs the flight-reproduction trade-off reported in some insects.

Base on Fig 2B and 2C, we reasoned that if *NlInR2*^E4^ had a same weight as *Wt*^SW^, the contents of glucose and triglyceride between these two types of BPHs would be even closer.

**Q2:***The authors focused on wings predominantly, for good reasons. But looking at the animals presented in Fig 1 D I immediately wonder what happened to other traits, including traits that already exist in nymphs (say eyes) and those that don't (like genitalia). If the authors want to hold this for another publication I can understand but if not it would make a possibly really nice addition*

**Respond to Q2:** *Nl*InR2 had hind tibia length (Fig 1D), head size (Fig 1E) and compound eyes (S2 Fig) comparable to *Wt*^SW^, **lines 170-172 in the combined manuscript_PG_clean copy**.

*NlInR2*^E4^ slightly and significantly reduced phallus length (Fig 1G), but had ovipositors with the size comparable to *Wt*^SW^ (Fig 1H), **lines 178-179 in the combined manuscript_PG_clean copy**. *NlInR2* null affect male genitalia, and a similar phenomenon was observed in the dung beetle *Onthophagus taurus* when the activity of *O. taurus* InR1 homologue was compromised by RNAi knockdown. We mentioned this in the discussion part, **lines 339-343 in the** **combined manuscript_PG_clean copy**.

**Q3:** *Fig 5: could the authors clarify if the go term analysis was contrasted to some kind of null expectation? As it is the results are used to support the hypothesis that InR2 regulates genes related to cell proliferation. Alternatively, this result could be expected by chance. Could the authors simulate what go term distributions they would get if drawing 657 genes at random from the BPH genome, then juxtapose that to their measured GO term frequencies?*

**Respond to Q3:** Sorry, we cannot fully understand the reviewer’s question. Anyway, we randomly picked 657 gene for GO analysis (**Rebuttal Letter Fig. 3**). This data showed GO terms different from Fig. 5C in the manuscript.

In addition, we did comparative transcriptome analysis of *Wt*^SW^, *NlInR2*^E4^, and *NlInR1*^RNAi^ females at 12 h after adult eclosion (**Rebuttal Letter Fig. 2A**; GenBank accession number: PRJNA724037). GO analysis of DEGs of *NlInR1*^RNAi^ vs *Wt*^SW^ and *NlInR2*^E4^ vs *Wt*^SW^ did not show a similar pattern as shown in Fig 5C.

Rebuttal Letter Fig. 3. GO term distributions of 657 genes randomly picked from the BPH genome.

**Q4:** *lines 270 onwards report expression changes in wing patterning genes following InR2 null mutations. This should probably be moved into the results section.*

**Respond to Q4:** We have moved this part into the results section, **lines 278-220 in the** **combined manuscript_PG_clean copy**.

**Q5:** *line 277: I agree that much more work is needed to examine InR paralog functions in other insects. In this section I would encourage the authors to cite and briefly discuss the work of Sofia Casasa on Onthophagus beetles published in Proceedings B. While she did not find strong evidence for functional divergence, she did examine both paralogs independently and I think that is worth mentioning. This then would allow the authors to explore two more points: (a) that sub/neofunctionalization may be lineage specific, possibly common in Hemiptera or Hemimetabola but not Holometabola and why that might be and (b) contrast Sofia's finding to their reference (22): InR RNAi affected horn formation in one subfamily of scarab beetles (Emlen et al) but the other (Casasa). Here already there is a solid hint that among subfamilies in teh same insect order InR function may diverge quite significantly.*

**Respond to Q5:** We provided a paragraph in the discussion section to discuss that the regulatory roles of multiple *InR*s on phenotypic plasticity might be insect lineage specific, and the work of Sofia Casasa on Onthophagus beetles was cited. **Please see lines 353-362 in the combined manuscript_PG_clean copy**.

MINOR COMMENTS
**Q6:***line 118: symmetrical patterning of wings: I suggest rewriting this and to focus on wing venation. When I read this I expected an analysis of symmetry, but the phenotypes that are described all focus on reduction in parts of the venation system*

**Respond to Q6:** InR2 null affected symmetrical vein patterning in wings. We have changed the interpretation and provided a new fig (Fig. 1I) for better showing that. We described this phenotype in **lines 182-189 and 348-352 in the combined manuscript_PG_clean copy**.

**Q7:** *line 158: ...increase wing cell number". Mentioning number specifically is important here I think because when I read that I thought wow specific cells in the wing get bigger*

**Respond to Q7:** “NlInR2-null mutants increase wing cells” was changed to “NlInR2-null mutants increase wing cell number” in the revised manuscript, **line 217 in the combined manuscript_PG_clean copy**.

**Q8:** *like 183: what is IFM? In general be mindful of overloading your reader with too many abbreviations. This one in particular though does not seem defined, at least I could not find it and after a while gave up so the hole section is lost on me.*

**Respond to Q8:** The full name of IFM first appeared in the introduction part (line 122). We used the full name (indirect flight muscles) in the revised manuscript.

WRITING SUGGESTIONS
The ms is very well written but on occasion some clarifications or changes in word choice might help. Here are some suggestion, please use what you like
**Q9:** *Line 1: Consider changing "Functionally independent evolution of..." to "Neofunctionalization of ..."*

**Respond to Q9:** The title has been changed to “Neofunctionalization of a second insulin receptor gene in the wing-dimorphic planthopper, *Nilaparvata lugens*”, as you suggested.

**Q10:***25: However, most insects possess additional copies of InR, yet the functional significance, if any, of alternate InRs is unknown*

**Respond to Q10:** We have changed the sentence as you suggested, **lines 25-26** **in the combined manuscript_PG_clean copy**.

**Q11:***30: "but differed greatly" to "but revealed distinct regulatory roles in"*

**Respond to Q11:** We have changed the sentence as you suggested, **lines 29-30** **in the combined manuscript_PG_clean copy**.

**Q12:** *48: such*

**Respond to Q12:** We have corrected the world, **line 48** **in the combined manuscript_PG_clean copy**.

**Q13:** *54: "possesses" instead of "had"*

**Respond to Q13:** We have corrected the world, **line 54** **in the combined manuscript_PG_clean copy**.

**Q14:***74: "in higher organisms" to "in mice and humans".*

**Respond to Q14:** We have changed the sentence, **line 73** **in the combined manuscript_PG_clean copy**.

**Q15:***56: ...may have functionally diversified in ways more complex than..*

**Respond to Q15:** We have changed the sentence, **line 56** **in the combined manuscript_PG_clean copy**.

**Q16:***123: "null mutants develop into viable LW morphs" also consider not using too many abbreviations in titles or subtitles*

**Respond to Q16:** We have changed the sentence, **line 154** **in the combined manuscript_PG_clean copy**. We also used full names in titles and subtitles in the revised manuscript.

**Q17:***152: ...females showed a ~27% reduction in fecundity...*

**Respond to Q17:** We have changed the sentence, **lines 201-203** **in the combined manuscript_PG_clean copy**.

**Q18:**195: *These results*

**Respond to Q18:** We have changed the world as you suggested, **line 255** **in the combined manuscript_PG_clean copy**.

**Q19:** *221: our understanding of their functions remains limited*

**Respond to Q19:** We have changed the world as you suggested, **lines 295-297** **in the combined manuscript_PG_clean copy**.

**Q20:** *268: To gain insights OR To look for insights*

**Respond to Q20:** The sentence was changed to “To gain insights into how…”, **line 285** **in the combined manuscript_PG_clean copy**.

**Respond to reviewers #3:**

*Reviewer #3: The manuscript describes studies that assess whether roles identified for an insulin receptor (InR1) in previous work are shared or differ for a second insulin receptor (InR2) encoded in the genome of the brown plant hopper (BPH). Using the CRISPR/Cas9 system, the authors disabled expression of InR2 in BPH and found that its absence was not lethal and favored development of long winged adults, unlike the control and InR1 RNAi treated individuals that became short-winged adults. InR2 mutants also took longer to develop and had reduced fecundity relative to control or InR1 RNAi individuals. Other related aspects of life history, metabolism, and gene expression were also examined and compared for the different treatment groups, either produced here or in previous work. The experimental design is appropriate, and the manuscript is well written and organized with lots of data for different but related biological aspects. The results and inferences are based on the phenotypic outcome of reverse genetics solely but do provide important insight into possible differences in InR1 and InR2 regulation of developmental and physiological processes in this up and coming model insect.*

Below are questions or suggestions for the authors to address.

**Q1:** *- Comparison/contrast of the pleiotropic regulation extensively characterized for the single InR in Drosophila and C. elegans to what little is known for the two InRs in BPH is repetitive and largely meaningless. More background information should be provided about other insect groups with two or more InRs and what has been discerned about their expression, signaling, and function. More relevant would be an examination of the extensive literature on the endocrine regulation of long/short wing cricket morphs and other hemimetabolous insects more closely related to BPH than Drosophila, which is a highly derived insect anomaly. More information especially about the structural and functional similarity of two InRs in BPH and Pyrrhocoris is warranted.*

**Respond to Q1:** (1) In the introduction section, we added more information to introduce multiple InRs paralogues and/or their functions in insect species including the rhinoceros beetles *Trypoxylus dichotomus*, damp-wood termite *Hodotermopsis sjostedti*, linden bug *Pyrrhocoris apterus*, honey bee *Apis mellifera*, bumblebee *Bombus terrestris*, fire ant *Solenopsis invicta*, green lacewing *Chrysopa pallens*, red flour beetle *Tribolium castaneum*, and brown citrus aphid *Aphis* (*Toxoptera*) *citricidus*. Please see **lines 88-97 and 102-117 in the** **combined manuscript_PG_clean copy**.

(2) In the introduction section, we added more information to introduce the endocrine regulation of long/short wing cricket, planthopper, and aphid morphs. Please see **lines 122-129 in the** **combined manuscript_PG_clean copy**.

(3) The sequence structural of InRs in BPH and Pyrrhocoris was compared in S1 Fig (lines 155-156 in the combined manuscript_PG_clean copy).

(4) The functional similarity and discrepancy of two InRs in BPH and Pyrrhocoris was introduced in lines 104-108 and 353-359, in the combined manuscript_PG_clean copy.

**Q2:***- A bit more context should be provided in the Introduction or Discussion about the number and type of insulin-like peptides, ligands for the InRs, and relevant downstream signaling elements encoded in BPH that may differentially affect the regulation/function of the two InRs.*

**Respond to Q2:** We introduced the insulin signaling elements in the introduction part of the revised manuscript. Please see lines 131-142 in the combined manuscript_PG_clean copy.

**Q3:***- Efficacy of the InR1 RNAi should be shown for the experimental sets described in this work. The same should be shown for the CRISPR/Cas9 InR2 mutants – are any full length InR2 transcripts detected by PCR in whole insects? Perhaps it is not completely disabled in all tissues, which may affect the interpretation of the results.*

**Respond to Q3:** We have provided the data to show the efficacy of the InR1 RNAi (S3 Fig), also see lines175-176 and 246-247 in the combined manuscript_PG_clean copy.

A “frame-shift” sequence of *NlInR2* transcripts could be detected by PCR, and the PCR products were directly used for Sanger sequencing or cloned for sequencing. The sequencing results confirmed that homozygous *NlInR2* null mutants were generated.

**Q4:**- *The GenBank accession number should be provided for the InR1.*

**Respond to Q4:** We added the GenBank accession number of *NlInR1* in the data availability section, lines 540-542 in the combined manuscript_PG_clean copy.

**Q5:***- There is no explanation in the Methods section (lines 347-354) how “relative cell number” was obtained/converted for the data points in Fig. 3B from the Ultrabithorax qRT-PCR data. Some procedure/formula with a reference is needed.*

**Respond to Q5:** Because the Hox gene *Ultrabithorax* is a single copy gene in the BPH genome, we used the copy number of *Ultrabithorax* to quantify gDNA copy number in qRT-PCR assay.

**Q6:**- *The punctuation or sentence structure in line 189 should be changed to separate the mutant annotations for better understanding of what is being conveyed.*

**Respond to Q6:** “*NlInR2*^E4^;ds*Gfp*” was replaced by “knockdown of *NlInR1* in *NlInR2*^E4^” in the revised manuscript, lines 248-250 in the combined manuscript_PG_clean copy.

**Q7:***- In the Discussion, expression of genes involved in wing patterning is compared for InR2 mutant and wild BPH – lines 262-276. The data presented in S1 Fig appear to be good enough and provide depth to the work and should be included in the Results in my opinion.*

**Respond to Q7:** We have moved this part into the results section, lines 278-290 in the combined manuscript_PG_clean copy.

**Q8:**- *Line 29 – “NlInR2 phenocopied…” makes no sense. Instead of using “phenocopied” (is it even a real word?), provide a more complete description that will aid the reader’s understanding.*

**Respond to Q8:** We used “resemble” instead of “phenotype” in the whole text.

**Q9:**- *What defines a “canonical” InR in any organism? Don’t think it is an appropriate modifier any place used in the text.*

**Respond to Q9:** Thank you for your suggestion. People usually say the canonical insulin signal pathway, but not canonical InR. We have removed “canonical” before InR in the revised manuscript.

**Q10:***- Line 78 – “two types of receptors are unified into a single InR in the fly” – there is no evidence for this especially since the single InR in the fly was there long before the InR and IGFR showed up in vertebrates.*

**Respond to Q10:** We have changed the sentence to be “However, a single *InR* but not *Igf-1r* was identified in the fly…”, lines 77-78 in the combined manuscript_PG_clean copy.

**Q11:***- Line 146 – “bona fide” is an adjective or noun and not used correctly here.*

**Respond to Q11:** We used “authentically” instead of “bona fide” in the revised manuscript, line193 in the combined manuscript_PG_clean copy.

**Q12:**- *Line 158 and another place – “To look insight into…” – drop “insight” or reword as “to gain insight”.*

**Respond to Q12:** “To look insight into…” was changed to “To look into…”, line 217 in the combined manuscript_PG_clean copy.

**Q13:**- Lines 203 – 215 – The discussion of the results is repetitive and confusing.

**Respond to Q13:** According to the PDF file merged by PG journal, lines 203 – 215 are in the paragraph of “*NlInR2*-null mutants temporally up-regulate genes associated with DNA replication in wings of 5^th^-instar nymphs” of the results section.

Please re-specify the sentences in our re-submitted manuscript so that we can revise them if we have a second round revision.

**Q14:**- *The title is too vague and should include information about the organism.*

**Respond to Q14:** The title has been changed to “Neofunctionalization of a second insulin receptor gene in the wing-dimorphic planthopper, *Nilaparvata lugens*”, as suggested by reviewer #2.
