# Supplementary material for: Neofunctionalization of a second insulin receptor gene in the wing-dimorphic planthopper, Nilaparvata lugens
Source: PLoS Genet. 2021 Jun 28;17(6):e1009653. doi: 10.1371/journal.pgen.1009653 (PMC8270448; doi:10.1371/journal.pgen.1009653)
Supplement: Attachment — Submitted filename: Response Letter.docx [file pgen.1009653.s029.docx]

**Point-to-point respond to reviewers**

**Q1：** Reviewer's Responses to Questions

**Comments to the Authors:**

**Please note here if the review is uploaded as an attachment.**

*Reviewer #1: The questions from previous review are addressed well. I think the data in the manuscript and additional experiments listed in response to review are convincing enough to show that NlInR1 and NlInR2 have distinct functions in BPH life history aspect. Experiments listed in response to review, especially the RNAseq data from adult female (Experiment #2) can be included in the supplementary file, unless the author want to use the data for other publication. It may not be the scope of this manuscript, but I think the point in line 328 (NlInR2 may serve as a negative regulator of NlInR1, as proposed previously [46]) is an important hypothesis worth testing. If there is any BPH cell line available, and if increased phosphorylation of NlAkt can be achieved by overexpressing NlInR1, an experiment can be designed to co-express NlInR1 and NlInR2 to see whether there is decrease in P-NlAkt compared to NlInR1 overexpression only. Since in reference [46], it is shown that NlInR and NlInR2 can bind each other [46: Figure2H], checking whether overexpressed NlInR2 can inhibit activity of NlInR1 mediated downstream insulin signaling can lead to interesting finding.*

**Respond to Q1:** In the revised manuscript, the RNAseq data from adult female was included in the supplementary file (S2 Data, S16-19 Tables, and S5 Fig), which was also cited in the main text (lines 335-339 in the manuscript_PG_clean copy).

Currently, there is no BPH cell line available. Actually, we did similar experiments reviewer proposed six year ago. We tried to expressed *NlInR1* and *NlInR2* in *Drosophila* S2 cells, and then measure P-Akt to read out the activity of insulin signaling pathway. However, we could not see any change of P-Akt level whether cells were expressed with *NlInR1* or *NlInR2* compared to cells alone.

**Q2：***Below are some minor suggestions.*

*In line 183, wing veins in forewings and hindwings, leading to a single individual with different wing patters at both sides of its body.*

**Respond to Q2:** We have revised the word in the revised manuscript, line 186 in the manuscript_PG_clean copy.

**Q3：***In 213*,*.. our findings indicate that NlInR2 resembles …*

**Respond to Q3:** We have revised the sentence in the revised manuscript, line 219 in the manuscript_PG_clean copy.

**Q4：***In 257, NlInR1 knockdown could antagonize the effects of NlInR2 knockout on wing and indirect flight muscles development*

**Respond to Q4:** We have revised the sentence in the revised manuscript, lines 261-262 in the manuscript_PG_clean copy.

**Q5：***In 282, morphogens hh, dpp, and wg*

**Respond to Q5:** We have revised the sentence in the revised manuscript, lines 286-287 in the manuscript_PG_clean copy.

**Q6：**Reviewer #2: *I think the authors have done a thorough and thoughtful set of revisions, addressing all my comments and I think most of those of the other reviewers. My only remaining suggestion would be to redo the statistical analysis of the effects of InR knockdowns on fecundity and other traits. Rather than using pairwise comparisons I think the authors should use a simple analysis of variance to be able to describe how much of e.g. changes in fecundity can be attributed to body size alone and how much to treatment. Right now the authors are guessing that some of these effects are negligible, this way they would know for sure. Further, they would be able to identify possible interaction effects, which right now they have no way of seeing. Any statistical consulting center should be able to help with this. Other than that minor issue the ms is in fine shape in my opinion.*

**Respond to Q6:** Reviewer #2 raised an important question on the correlation coefficient of trait size and body size. There are two points we have to make.

(1) Unlike some big insects, BPHs are tiny (< 5 mm in body length) and light (~2 mg), so we usually don’t weigh its body mass individually. In other words, big STEDV will be produced when you weigh BPH individuals, even if using an analytical balance. Instead, we usually used hind tibia length to reflect its body size. The tiny body size also impaired us to measure glucose and lipid level individually. That’s the reason why we used pooled sample for body weight (Fig 2B), glucose (Fig 2C), and triglyceride (Fig 2D). A similar method was also used in *Drosophila* studies.
(2) We re-analyzed the size of forewing, phallus, and ovipositor, and fecundity by using hind tibia length to represent its body size. We found that the correlation coefficients (R^2^) of wing size, phallus length, ovipositor length, and egg numbers relative to its hind tibia length in *Wt*^LW^, *Wt*^SW^, or *NlInR2*^E4^ BPHs were no more than 0.13 (Fig 1F, 1G, 1H, and Fig 2E), indicating that the sizes in these tissues were not correlated with the body size (lines 179-183 and 208-211 in the manuscript_PG_clean copy).

**Q7：**Reviewer #3: The revised manuscript is much improved and a clearer case made for this important contribution. The authors satisfactorily addressed all of my suggestions and concerns.

**Respond to Q7:** Thank you for your reviewing.
